# Differential Regulation of Bilastine Affinity for Human Histamine H_1_ Receptors by Lys 179 and Lys 191 via Its Binding Enthalpy and Entropy

**DOI:** 10.3390/ijms22041655

**Published:** 2021-02-06

**Authors:** Hayato Akimoto, Minoru Sugihara, Shigeru Hishinuma

**Affiliations:** 1 Department of Pharmacodynamics, Meiji Pharmaceutical University, 2-522-1 Noshio, Kiyose, Tokyo 204-8588, Japan; d206901@std.my-pharm.ac.jp; 2 Pharmaceutical Education and Research Center, Meiji Pharmaceutical University, 2-522-1 Noshio, Kiyose, Tokyo 204-8588, Japan; sugihara@my-pharm.ac.jp

**Keywords:** affinity, antihistamine, bilastine, enthalpy, entropy, histamine H_1_ receptor

## Abstract

Bilastine, a zwitterionic second-generation antihistamine containing a carboxyl group, has higher selectivity for H_1_ receptors than first-generation antihistamines. Ligand-receptor docking simulations have suggested that the electrostatic interaction between the carboxyl group of second-generation antihistamines and the amino group of Lys179^ECL2^ and Lys191^5.39^ of human H_1_ receptors might contribute to increased affinity of these antihistamines to H_1_ receptors. In this study, we evaluated the roles of Lys179^ECL2^ and Lys191^5.39^ in regulating the electrostatic and hydrophobic binding of bilastine to H_1_ receptors by thermodynamic analyses. The binding enthalpy and entropy of bilastine were estimated from the van ’t Hoff equation using the dissociation constants. These constants were obtained from the displacement curves against the binding of [^3^H] mepyramine to membrane preparations of Chinese hamster ovary cells expressing wild-type human H_1_ receptors and their Lys179^ECL2^ or Lys191^5.39^ mutants to alanine at various temperatures. We found that the binding of bilastine to wild-type H_1_ receptors occurred by enthalpy-dependent binding forces and, more dominantly, entropy-dependent binding forces. The mutation of Lys179^ECL2^ and Lys191^5.39^ to alanine reduced the affinity of bilastine to H_1_ receptors by reducing enthalpy- and entropy-dependent binding forces, respectively. These results suggest that Lys179^ECL2^ and Lys191^5.39^ differentially contribute to the increased binding affinity to bilastine via electrostatic and hydrophobic binding forces.

## 1. Introduction

**Figure 1:**
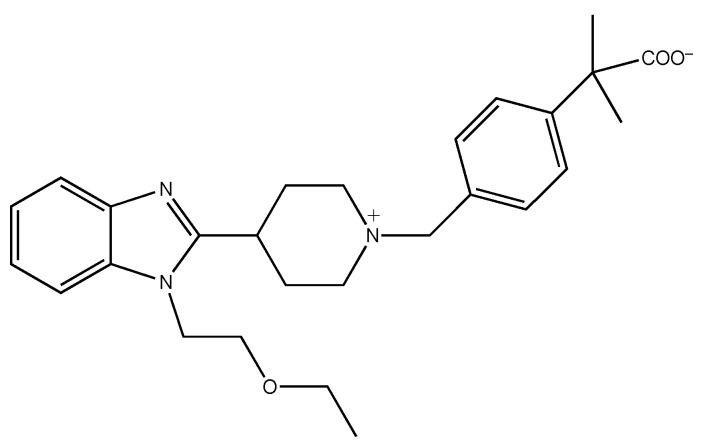
Chemical structure of bilastine. Bilastine is a zwitterionic second-generation antihistamine containing a carboxyl group that contributes to its non-sedative properties as well as an increased selectivity for H_1_ receptors.

It is known that G_q/11_-protein-coupled H_1_ receptors are involved in mediating allergic and inflammatory responses in peripheral tissues and the state of arousal in the central nervous system [1,2,3,4,5,6,7,8]. Various first- and second-generation antihistamines have been developed for the treatment of type I hypersensitivity, such as allergic rhinitis [9,10,11,12,13]. Some second-generation antihistamines have zwitterionic properties owing to the presence of carboxyl groups, which help reduce their side effects such as sedation and impaired performance resulting from the blockade of H_1_ receptors in the central nervous system via their penetration into the brain through the blood–brain barrier. Bilastine (Figure 1), a zwitterionic second-generation antihistamine, has non-sedative properties as well as an increased selectivity for H_1_ receptors than first-generation antihistamines [13,14,15,16,17,18,19].

**Figure 2:**
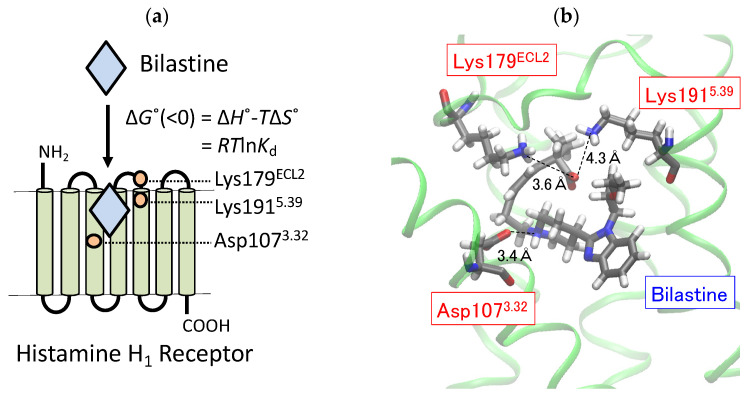
A schematic structure of human H_1_ receptor (**a**) and docking simulation on the binding of bilastine to human H_1_ receptor (**b**). (**a**) A schematic structure of human H_1_ receptor is shown and indicates that Asp107^3.32^ is a highly conserved amino acid in the aminergic G-protein-coupled receptors, and Lys179^ECL2^ and Lys191^5.39^ are anion-binding sites unique to the H_1_ receptor. Ligand-receptor interaction is also shown to demonstrate that the binding affinity for ligands (*K*_d_) is determined by their thermodynamic binding forces (∆*G*° = ∆*H*° − *T*∆*S*° = *RT*ln*K*d). (**b**) Docking simulation was performed to reveal the final position of bilastine binding to human H_1_ receptor as described in the materials and methods section. The amino group of bilastine appeared to interact with Asp107^3.32^, and the carboxyl group of bilastine appeared to interact with Lys179^ECL2^ and Lys191^5.39^. Atoms of nitrogen, oxygen, carbon, and hydrogen are shown in blue, red, gray, and white, respectively. The distances between key atoms of bilastine and amino acid residues are also shown.

The human H_1_ receptor possesses Asp107^3.32^ (superscripts indicate Ballesteros-Weinstein numbering [20]), a highly conserved amino acid in aminergic G protein-coupled receptors, deep in the ligand-binding pocket, whereas Lys179^ECL2^ and Lys191^5.39^, located at the entrance of the ligand-binding pocket, are anion-binding sites unique to the H_1_ receptor (Figure 2a) [21]. Ligand-receptor docking simulations based on the crystal structure of human H_1_ receptor indicated that the carboxyl group of second-generation antihistamines, such as olopatadine, levocetirizine, fexofenadine, and acrivastine, appeared to form a salt bridge with Lys179^ECL2^ and/or Lys191^5.39^. This bridge might have contributed to the increased selectivity of carboxylated second-generation antihistamines for H_1_ receptors [21]. Accordingly, the electrostatic interaction of bilastine with Lys179^ECL2^ and/or Lys191^5.39^ may be important in the determination of its binding affinity for H_1_ receptors.

The binding affinity (*K*_d_) of ligands is determined by the thermodynamic binding forces of ligands, and these are the binding enthalpy (∆*H*°) and entropy (∆*S*°) (Figure 2a) [22,23,24,25,26,27]. ∆*H*° is usually associated with binding forces via the formation of new bonds between receptors and ligands, such as electrostatic interaction via salt bridges, hydrogen bonds and van der Waals interactions, whereas ∆*S*° is usually characterized by binding forces via the displacement of ordered water molecules coupled with the formation of new hydrophobic interactions [22,23,24,25,26,27,28,29,30]. Thus, thermodynamic analyses provide important information for evaluating the electrostatic and hydrophobic binding of bilastine with H_1_ receptors. In this study, we examined how Lys179^ECL2^ and Lys191^5.39^ might increase the electrostatic and hydrophobic binding of bilastine with H_1_ receptors through the mutation of Lys179^ECL2^ and/or Lys191^5.39^ to alanine.

## 2. Results and Discussion

### 2.1. Docking Simulation on the Binding of Bilastine to H_1_ Receptor

Docking simulation was performed to simply estimate the configuration of bilastine at the ligand-binding pocket of human H_1_ receptors (Figure 2b). We found that the carboxyl group of bilastine was located between Lys179^ECL2^ and Lys191^5.39^ and that the amino group of bilastine was close to Asp107^3.32^. The oxygen atom of the carboxyl group of bilastine appeared to be closer to the nitrogen atom of Lys179 (3.6 Å) than that of Lys191 (4.3 Å). Since the docking simulation could not exactly predict roles of Lys179^ECL2^ and Lys191^5.39^ in regulating the binding affinity of bilastine, we then performed receptor binding experiments using [^3^H] mepyramine, a radioligand for H_1_ receptors, to evaluate actual roles of Lys179^ECL2^ and Lys191^5.39^ in regulating the binding affinity of bilastine via its thermodynamic binding forces.

### 2.2. Roles of Lys179^ECL2^ and Lys191^5.39^ in the Binding Affinity for Bilastine

**Figure 3:**
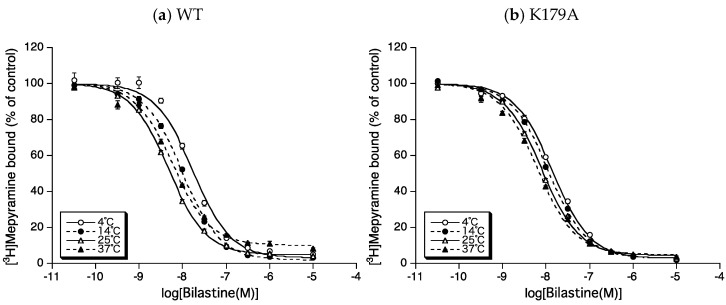

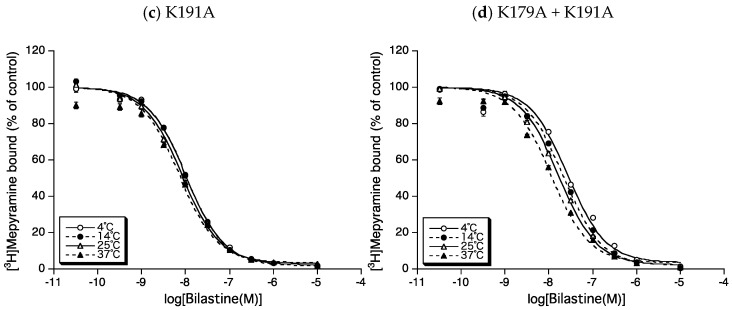
Displacement curves for bilastine against the binding of [^3^H] mepyramine to WT (**a**), K179A (**b**), K191A (**c**), and K179A + K191A (**d**). The binding of 3 nM [^3^H]mepyramine to membranes containing WT (**a**), K179A (**b**), K191A (**c**), and K179A + K191A (**d**) in the presence or absence of various concentrations of bilastine was measured at 4 °C (open circles), 14 °C (closed circles), 25 °C (open triangles), and 37 °C (closed triangles), as described in materials and methods section. The data points are the percentages of bound [^3^H] mepyramine, with 100% as 3 nM [^3^H] mepyramine binding in the absence of bilastine. Data represent mean ± standard errors of means of 4–7 independent experiments determined in quadruplicates. The lines are the best-fit curves to a one-site model.

To evaluate changes in the binding affinity of bilastine for H_1_ receptors by mutations of Lys179^ECL2^ and/or Lys191^5.39^, the *IC_50_* for bilastine was first obtained from the displacement curves for the binding of 3 nM [^3^H] mepyramine to membrane preparations of CHO cells expressing wild-type human H_1_ receptors (WT), Lys179^ECL2^ or Lys191^5.39^ mutants of WT to alanine (K179A and K191A), and both Lys179^ECL2^ and Lys191^5.39^ mutants of WT to alanine (K179A + K191A) at 4 °C–37 °C (Figure 3). The *K*_i_ values for bilastine were then calculated from the *IC_50_*, as described in the materials and methods section.

**Figure 4:**
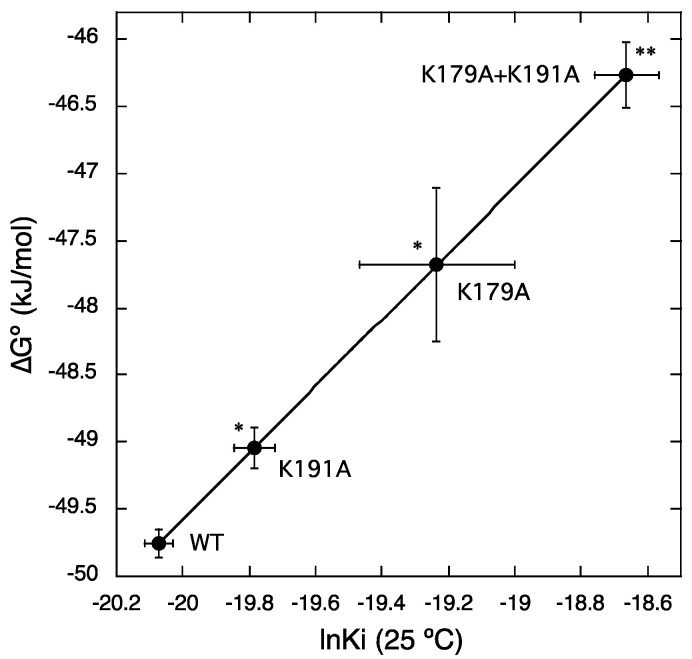
Changes in the values of ∆*G*° and ln*K*_i_ of bilastine by mutations in Lys179^ECL2^ and/or Lys191^5.39^. Scatter plots of values of ∆*G*° versus ln*K*_i_ of bilastine for WT, K179A, K191A, and K179A + K191A are shown, in which ∆*G*° values were calculated according to the equation, ∆*G*° = *RT*ln*K*i, at a standard temperature of 25 °C (298.15 K). Increases in values of ∆*G*° and ln*K*_i_ represent reductions in the affinities for bilastine by mutations of Lys179^ECL2^ and/or Lys191^5.39^. * *p* < 0.05, ** *p* < 0.01; compared to the values for WT.

Figure 4 shows the ln*K*_i_ values of bilastine and their corresponding ∆*G*° values (∆*G*° = *RT*ln*K*_i_) for WT, K179A, K191A, and K179A + K191A at a standard temperature of 25 °C (298.15 K). The *K*_i_ values of bilastine were 1.92 ± 0.08 nM for WT (*n* = 4), 5.20 ± 1.18 nM for K179A (*n* = 7), 2.57 ± 0.16 nM for K191A (*n* = 4), and 7.96 ± 0.76 nM for K179A + K191A (*n* = 4). Thus, the affinity of bilastine for H_1_ receptors was significantly reduced by approximately 1.3 to 4.1 times following the mutation of Lys179^ECL2^ and/or Lys191^5.39^. These results suggest that Lys179^ECL2^ and Lys191^5.39^ contribute to the increased affinity for bilastine.

### 2.3. Roles of Lys179^ECL2^ and Lys191^5.39^ in Thermodynamic Binding Forces of Bilastine

**Figure 5:**
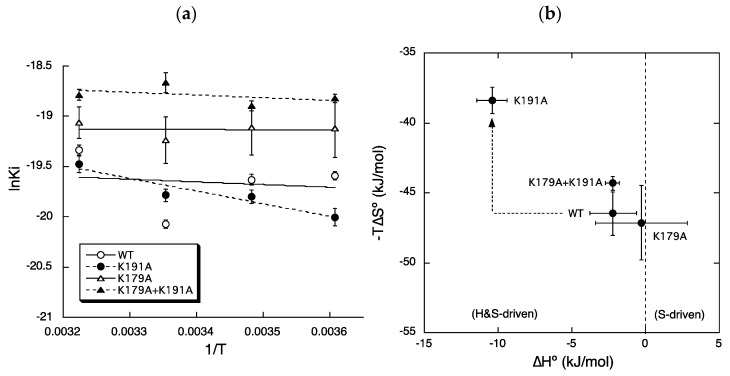
Changes in the thermodynamic binding forces of bilastine by mutations of Lys179^ECL2^ and/or Lys191^5.39^. (**a**) van ‘t Hoff plots for bilastine: according to the van ‘t Hoff equation, ln*K*_i_ = ∆*H*°/*RT* − ∆*S*°/R, the slope and intercept of the vertical axis represent ∆*H*°/*R* and −∆*S*°/*R*, respectively. (**b**) Scatter plots of values of −*T*∆*S*° versus ∆*H*°: compounds with a negative value of ∆*H*° and positive value of −*T*∆*S*° are classified as enthalpy driven (*H* driven); conversely, compounds with a positive value of ∆*H*° and negative value of −*T*∆*S*° are classified as entropy driven (*S* driven). Compounds with negative values of ∆*H*° and −*T*∆*S*° are classified as enthalpy and entropy driven (*H*&*S* driven). Reductions in values of ∆*H*° and −*T*∆*S*° reveal increases in the binding forces of ligands mediated by enthalpy and entropy, respectively. The arrow indicates changes induced by the mutation of Lys191 to alanine.

Figure 5a shows the van ’t Hoff plots used to determine the thermodynamic binding forces of bilastine according to the equation, ln*K*_i_ = ∆*H*°/*RT* − ∆*S*°/*R*. Figure 5b shows scatter plots of values of −*T*∆*S*° versus ∆*H*° for bilastine obtained from the van ’t Hoff plots. In the binding of bilastine to WT (Figure 5b; WT), negative values of ∆*G*° (= ∆*H*° − *T*∆*S*°) for bilastine were obtained by the binding enthalpy (∆*H*°) and more dominantly the binding entropy (−*T*∆*S*°). These results are consistent with our previous findings that entropy-dependent binding forces of second-generation antihistamines were significantly higher than those of first-generation antihistamines [25].

The mutation of Lys179^ECL2^ to alanine (Figure 5b; K179A) led to a reduction in the enthalpy-dependent binding forces (∆*H*°) of bilastine by 1.9 kJ/mol although not significantly. This might explain the reduction in the affinity of bilastine by the mutation of Lys179^ECL2^ to alanine. Thus, Lys179^ECL2^ may have played a role in maintaining the electrostatic binding forces of bilastine.

The mutation of Lys191^5.39^ to alanine (Figure 5b; K191A) led to a marked change in the binding enthalpy and entropy of bilastine compared with the expected. The entropy-dependent binding forces (∆*T*∆*S*°) of bilastine were significantly reduced by 8.2 kJ/mol, which may explain the reduced affinity of bilastine following mutation. Thus, Lys191^5.39^ may play a crucial role in maintaining the hydrophobic binding forces of bilastine. Conversely, enthalpy-dependent binding forces (∆*H*°) of bilastine were significantly increased by 8.1 kJ/mol due to the mutation of Lys191^5.39^. Thus, Lys191^5.39^ may play an inhibitory role in the electrostatic binding of bilastine. These results are in agreement with our findings that Lys191^5.39^ might not necessarily contribute to electrostatic binding forces of carboxylated antihistamines such as levocetirizine [26]. It should be noted that Lys179^ECL2^ and Lys191^5.39^ differentially regulated the enthalpy- and entropy-dependent binding forces of bilastine.

The mutation of both Lys179^ECL2^ and Lys191^5.39^ to alanine led to a reduction in the entropy-dependent binding forces (∆*T*∆*S*°) of bilastine by 2.2 kJ/mol although not significantly, which might explain the reduction in the affinity of bilastine. It is most likely that Lys179^ECL2^ and Lys191^5.39^ interacted with bilastine at the entrance of the ligand-binding pocket of H_1_ receptors to increase the binding affinity of bilastine via electrostatic and hydrophobic interactions, as it is assumed that ligands may interact with both positively and negatively charged regions as well as hydrophobic transmembrane domains of the receptor before reaching the final position in the ligand-binding pocket [27].

In conclusion, the study revealed that the binding of bilastine to H_1_ receptors occurred by the binding enthalpy (∆*H*°) and the binding entropy (∆*T*∆*S*°) and that Lys179^ECL2^ and Lys191^5.39^ play a differential role in regulating the thermodynamic binding forces of bilastine. These findings provide further insight into the mechanisms by which the affinities of ligands for their receptors are individually regulated by electrostatic and hydrophobic interactions.

## 3. Materials and Methods

### 3.1. Materials

[Pyridinyl-5-^3^H]-mepyramine was purchased from PerkinElmer (Waltham, MA, USA). Bilastine was purchased from Tokyo Chemical Industry (Tokyo, Japan). Chinese hamster ovary cells (CHO-K1: RCB0285, RRID: CVCL_0214) were purchased from the RIKEN Bioresource Center (Tsukuba, Ibaraki, Japan). The expression vectors (3×HA hH1R/pcDNA3.1(+)) for human H_1_ receptors tagged with three molecules of hemagglutinin (YPYDVPDYA) at the N terminus were purchased from the Missouri S&T cDNA Resource Center (Rolla, MO, USA). Other materials were purchased from Sigma-Aldrich (Tokyo, Japan).

### 3.2. Docking Simulation on the Binding of Bilastine to Human H_1_ Receptor

A docking engine, Sievgene, implemented in MyPresto5.0 (N^2^PC, Tokyo, Japan) [31] was used in for the present study under the following settings: Flexible ligand, rigid protein, and no water molecule. The docking score was a modified version of the multiple active site correction score [32]. The ligand binding site was indicated by a set of reference points, which were the atom coordinates of the ligand in the target protein-ligand complex. The ligand atoms were superposed to the binding site using the geometric hashing method [33] and the optimal complex structure was obtained by using the steepest decent algorithm with the AMBER-type molecular force field. The interactions that accounted for this method were van der Waals, Coulomb, hydrogen bond, and hydrophobic interactions. In this study, the target protein model was generated from the crystal structure of the human H_1_ receptor (PDB:3RZE) [21]. Docking calculations were performed by placing bilastine in a random position within 6.5Å from the binding site and optimizing the steepest descent algorithm.

### 3.3. Measurement of [^3^H]Mepyramine Binding to Membrane Preparations

CHO cells stably expressing WT, K179A, K191A, and K179A + K191A were cultured, and membrane preparations were obtained as described previously [25,26,27]. The receptor binding assay with [^3^H] mepyramine, a radioligand for H_1_ receptors, was performed in accordance with the methods described previously [25,26,27]. Briefly, aliquots (0.1 mL) of membrane preparations (approximately 50 µg of membrane proteins) were incubated with 3 nM [^3^H] mepyramine in the presence or absence of various concentrations of bilastine for 3 h at 37 °C, 24 h at 25 °C and 14 °C, and 7 days at 4 °C in normal HEPES buffer (NaCl, 120 mM; KCl, 5.4 mM; MgCl_2_, 1.6 mM; CaCl_2_, 1.8 mM, D-glucose, 11 mM; and HEPES, 25 mM; pH 7.4 at 37 °C; final volume 1 mL). The reaction mixture was filtered through glass fiber filters, and the radioactivity trapped on the filters was determined by scintillation counting. All determinations were made in quadruplicate. The protein content in the membrane preparations was determined using a BCA protein assay kit (Pierce, Rockford, IL, USA).

### 3.4. Data Analyses

All data were presented as means ± standard errors of means of at least three measurements performed in quadruplicate. Statistical significance was evaluated using Student’s *t*-test or analysis of variance with Bonferroni correction. Results with a value of *p* < 0.05 were considered significant.

The *IC_50_* for bilastine was determined by fitting the displacement curves to the one-site model (KaleidaGraph; Synergy Software, Reading, PA, USA):

*B* = 100 − *P* × *C*/(*C* + *IC*_50_)

where *B* is the amount of [^3^H] mepyramine bound, *C* is the free concentration of bilastine, and *P* is the percentage of the binding sites of bilastine.

The *K*_i_ values for bilastine were estimated from the Cheng and Prusoff equation, as follows [25,26,27,34]:

*K*_i_ = *IC*_50_/(*C*/*K*_d_ + 1)

where *K*_i_ is the dissociation constant for bilastine, *C* is the free concentration of [^3^H] mepyramine, and *K*_d_ is the dissociation constant for [^3^H] mepyramine.

## Data Availability

Not applicable.

## Abbreviations

CHO	Chinese hamster ovary
K179A	Lys179 mutant of H_1_ receptor to alanine
K191A	Lys191 mutant of H_1_ receptor to alanine
K179A + K191A	Both Lys179 and Lys191 mutant of H_1_ receptor to alanine
WT	Wild-type H_1_ receptor
